# Hexavalent Chromium Exposure Induces Intestinal Barrier Damage *via* Activation of the NF-κB Signaling Pathway and NLRP3 Inflammasome in Ducks

**DOI:** 10.3389/fimmu.2022.952639

**Published:** 2022-07-22

**Authors:** Chenghong Xing, Fan Yang, Yiqun Lin, Jiyi Shan, Xin Yi, Farah Ali, Yibo Zhu, Chang Wang, Caiying Zhang, Yu Zhuang, Huabin Cao, Guoliang Hu

**Affiliations:** ^1^ Jiangxi Provincial Key Laboratory for Animal Health, Institute of Animal Population Health, College of Animal Science and Technology, Jiangxi Agricultural University, Nanchang, China; ^2^ Department of Theriogenology, Islamia University of Bahawalpur, Bahawalpur, Pakistan

**Keywords:** hexavalent chromium, intestinal barrier, NF-κB, NLRP3, duck

## Abstract

Hexavalent chromium [Cr(VI)] is a dangerous heavy metal which can impair the gastrointestinal system in various species; however, the processes behind Cr(VI)-induced intestinal barrier damage are unknown. Forty-eight healthy 1-day-old ducks were stochastically assigned to four groups and fed a basal ration containing various Cr(VI) dosages for 49 days. Results of the study suggested that Cr(VI) exposure could significantly increase the content of Cr(VI) in the jejunum, increase the level of diamine oxidase (DAO) in serum, affect the production performance, cause histological abnormalities (shortening of the intestinal villi, deepening of the crypt depth, reduction and fragmentation of microvilli) and significantly reduced the mRNA levels of intestinal barrier-related genes (ZO-1, occludin, claudin-1, and MUC2) and protein levels of ZO-1, occludin, cand laudin-1, resulting in intestinal barrier damage. Furthermore, Cr(VI) intake could increase the contents of hydrogen peroxide (H_2_O_2_) and malondialdehyde (MDA), tumor necrosis factor-α (TNF-α), interleukin-1β (IL-1β), and interleukin-18 (IL-18) but decrease the activities of total superoxide dismutase (T-SOD), catalase (CAT), and glutathione reductase (GR), as well as up-regulate the mRNA levels of TLR4, MyD88, NF-κB, TNFα, IL-6, NLRP3, caspase-1, ASC, IL-1β, and IL-18 and protein levels of TLR4, MyD88, NF-κB, NLRP3, caspase-1, ASC, IL-1β, and IL-18 in the jejunum. In conclusion, Cr(VI) could cause intestinal oxidative damage and inflammation in duck jejunum by activating the NF-κB signaling pathway and the NLRP3 inflammasome.

## Introduction

Chromium (Cr) is a crudely occurring element that can be found in diverse environmental media such as soils, water, air, and sediments (1, 2). It exists in an amount of oxidation states ranging from -2 to +6. Trivalent chromium [Cr(III)] and hexavalent chromium [Cr(VI)] are the most common valence states (3). Cr(III) is a relatively stable essential trace element that plays a critical role in human carbohydrate, lipid, and protein metabolism (4). Unlike Cr(III), Cr(VI) is highly mobile in soil and exceedingly hazardous to living creatures and is designated as a carcinogen by the World Health Organization (WHO) (5). With the widespread use of Cr(VI) in wood preservation, leather tanning, chrome plating, dye production, and alloy manufacturing, large amounts of Cr(VI) are mined/produced globally each year, resulting in environmental pollution (6–8). The total amount of Cr(VI) discharged in soil, water, and air globally per year is estimated to be 896,000, 142,000, and 30,000 metric tons, respectively (9), which is well above the international guideline (50–100 kg/year) (10). Cr(VI) has well-known solubility, mobility, and responsiveness that can easily enter animal and human bodies through the skin, gastrointestinal tract, or respiratory tract, thus leading to Cr(VI) poisoning (11). Previous studies have indicated that Cr(VI) exposure could induce immunotoxicity, dermatotoxicity, genotoxicity, neurotoxicity, and carcinogenicity in different tissues of animals and humans (12, 13).

The intestine not only is responsible for the digestion and absorption of nutrients but also is the largest immune organ that is closely related to the health of animals (14, 15). It plays a crucial role in resisting the invasion of pathogenic bacteria and the entry of xenobiotics (16). Intestinal environment homeostasis is maintained by the epithelial cell integrity, intestinal mucosal immunity, and complex interaction between the intestinal microbiota and nutrients (17, 18). Numerous investigations have shown that heavy metal exposure negatively impacts gut function (19, 20). Zhang et al. demonstrated that lead exposure in carp altered gut microbiota and destroyed intestinal structural integrity by inhibiting the expression of intestinal epithelial tight-junction proteins (21). Zhong et al. demonstrated that arsenic trioxide could cause jejunum inflammation *via* increasing the production of pro-inflammatory cytokines in ducks (22). Furthermore, Zhou et al. discovered that subchronic mercury exposure led to gut microbiota dysbiosis and metabolic disturbances in chickens (23). These studies illustrated that heavy metal exposure could induce intestinal toxicity by destroying the intestinal barrier in different ways. In recent years, many studies have also shown that long-term exposure to Cr(VI) could cause intestinal damage and adversely affect the intestinal immunity of chickens (24, 25). Nevertheless, the mechanism by which Cr(VI) induced intestinal toxicity remains unknown. Proverbially, oxidative stress is one of the most crucial mechanisms of Cr(VI)-induced tissue injury (26). When Cr(VI) enters the cell, it acts as a strong oxidant and can produce additional reactive oxygen species (ROS) (27). The body’s antioxidants are greatly reduced as ROS continues to accumulate. The cells are then attacked, resulting in cellular injury, which then produces inflammatory cytokines, which activate immune cells, resulting in inflammation that eventually causes tissue damage (28–32). A slew of recent studies has suggested that inflammation is linked to the activation of the NF-κB signaling pathway and the NLRP3 inflammasome (33, 34). However, the activation mechanism of Cr(VI)-induced intestinal inflammation remains uncertain. Therefore, the mechanism of Cr(VI)-induced intestinal barrier damage in ducks *via* activation of the oxidative stress-mediated NF-κB signaling pathway and the NLRP3 inflammasome needs to be further investigated.

Ducks are more susceptible to Cr(VI) toxicity than other animals due to ingestion of Cr(VI)-contaminated water and soil. Ducks better reflect the toxic effects of Cr(VI). Hence, ducks were used in this study to assist the researchers in better understanding the potential mechanisms of Cr(VI)-induced intestinal barrier damage and lay the groundwork for future research.

## Materials and methods

### Experiment Animals and Management

The experimental procedures were in favor of the animal ethics committee of Jiangxi Agricultural University (Approval ID: JXAULL-2022003). Ducks were purchased from Nanchang Miaowang Industrial Co., Ltd. (Nanchang, China), and fed with the standard rations as recommended by the National Research Council (NRC), which cited Ren et al. (35). **Table 1** displays the basic diet. The experimental animals were housed in a controlled environment with free access to water and a duck basal diet.

**Table 1:** The composition and nutritional levels of the basal diet.

Item (%, unless noted)	Content
Corn	47
Wheat bran	13
Rice bran	9
Soybean meal, 43%	9
Rapeseed meal	9
Cottonseed meal	6
Rapeseed oil	2.88
Calcium corbonate	0.96
Dicalcium plosphate, 2H_2_O	1.275
L-Lysine-HCl	0.37
D, L-Methionine	0.226
Threonine, 98.5%	0.044
Tryptophan, 98.5%	0.032
Sodium chloride	0.4
Choline chloride, 50%	0.2
Bentonite	0.913
Mineral premix1	0.4
Vitamin premix2	0.2
Analyzed nutrient content	
ME (Kcal/kg, calculated)	2914
CP (analyzed)	17.12
Calcium (analyzed)	0.94
Total phosphorus (analyzed)	0.84
Nonphytate phosphorus (calculated)	0.478

^1^Dietary supply per kilogram: copper, 8 mg; iron, 80 mg; zinc, 90 mg; manganese, 70 mg; selenium, 0.3 mg; iodine, 0.4 mg.

^2^Dietary supply per kilogram: vitamin A, 15,000 IU; vitamin D_3_, 5000 IU; vitamin K_3_, 5 mg; vitamin E, 80 mg; vitamin B_1_, 3 mg; vitamin B_2_, 9 mg; vitamin B_6_, 7 mg; vitamin B_12_, 0.04 mg; nicotine acid, 80 mg; pantothenic acid, 15 mg; biotin, 0.15 mg; folic acid, 2 mg; vitamin C, 200 mg; 25-hydroxycholecalciferol, 0.069 mg.

### Determination of Lethal Dose 50 (LD_50_)

The source of Cr(VI) was analytical-grade potassium dichromate (K_2_Cr_2_O_7_) (Sigma-Aldrich, St. Louis, CA, USA, 99% purity). For the determination of LD_50_, 16 healthy Tianfu meat ducks (1 day old) were stochastically assigned to four groups of four ducks each. Different doses of Cr(VI) were administered to these groups that were infected *via* oral gavage, namely, 0.215, 0.464, 1.0, and 2.15 g/kg body weight. The gavage volume was calculated as 1% of body weight, fasting before gavage lasted 6 h, drinking water was not restricted, and the observation lasted 14 days. According to the Horn Table (the People’s Republic of China’s national standard acute toxicity test), the LD_50_ of K_2_Cr_2_O_7_ in ducks was 0.464 g/kg, with a 95% confidence limit of 0.298–0.723 g/kg.

### Toxicity Trials and Sample Collection

Before the experiment, 48 Tianfu meat ducks (1 day old) were housed in an appropriate environment for 7 days. Then ducks were divided into four groups and given Cr(VI) in doses of 0 (Control), 9.28 (LCr), 46.4 (MCr), and 232 (HCr) mg/kg body weight for 49 days. All ducks were weighed, and blood samples were collected from each duck’s wing vein and separated serum on day 49. The jejuna were removed from each group of 12 ducks promptly after euthanasia. The length and weight of the jejunum tissues were measured. Then, the jejunum was cut into small pieces and rinsed repeatedly with 0.9% NaCl until there was no stool. A part of jejunum specimens was stored at -20°C for determining contents of Cr(VI), and a part of jejunum specimens was fixed in 10% formalin and 2.5% glutaraldehyde for histopathological and ultrastructural observation, and the rest of the jejuna were stored at -80°C for RNA isolation and total protein extraction.

### Histopathological Examination

The jejunum tissues were fixed in 10% formalin for 24 h, then dehydrated, embedded, sliced, and stained for microscopy (36). TissueFAXS was applied to measure and analyze villus length and crypt depth (TissueFAXS Plus, Vienna, Austria).

The effect of Cr(VI) on jejunum tissues was studied using periodic acid–Schiff (PAS) staining. PAS staining was performed according to the instructions provided by the manufacturer of the PAS Kit (Cat # G1049, Service, Wuhan, China). TissueFAXS was used to count the number of goblet cells in all of the sections (TissueFAXS Plus, Austria).

### Transmission Electron Microscopy

Transmission electron microscopy (TEM) was carried out in accordance with the protocol previously described (37, 38). After collecting jejunum tissues, they were processed and examined using a TEM HT7800 (Hitachi, Tokyo, Japan). The ultrastructural pathological changes in the jejunum were observed and compared to the control group counterparts.

### Determination of Cr(VI) Contents in Jejunum Tissues

The jejunum tissues were digested with HNO_3_ (65%) and H_2_O_2_ (30%) before being diluted to 10 ml with deionized water. Following that, the samples were digested and analyzed using a microwave system and inductively coupled plasma mass spectrometry (ICP-MS) (NexION 350, Watertown, MA, USA).

### Determination of Diamine Oxidase and Oxidative Stress Index Content

Commercially available diagnostic kits (Jiancheng Biotech, Nanjing, China) were applied to detect the concentrations of diamine oxidase (DAO) in serum. Additionally, H_2_O_2_ and MDA concentrations and T-SOD, CAT, and GR activities in jejunum tissues were also detected as previously described (39).

### Determination of Inflammation Cytokines

TNF-α, IL-1β, and IL-18 levels were detected in jejunum tissues using a commercial kit (mlbio, Shanghai, China). All operation steps were carried out according to the manual.

### Real-Time Quantitative PCR

Total RNA was isolated from jejunum tissues using TRIzol reagent (Takara, Shiga, Japan) and reverse transcribed, as directed by the manufacturer. Then, the reverse transcription product was used for RT-PCR. The gene sequences for duck ZO-1, occludin, claudin-1, MUC2, TLR4, MyD88, NF-κB, TNFα, IL-6, NLRP3, caspase-1, ASC, IL-1β, IL-18, and GAPDH are shown in **Table 2**. Primers were designed with Primer Express 3.0 software and synthesized by Beijing Qingke Biotechnology Co., Ltd. The experiment was carried out on a CFX384 Touch+CFX PCR instrument (Bio-Rad, Hercules, CA, USA). Pre-denaturation was at 95°C for 30 s, denaturation at 95°C for 5 s, annealing at 60°C for 34 s, 40 cycles. The data were normalized to GAPDH expression and analyzed using the 2^-△△^CT method.

**Table 2:** Premier sequences used for real-time PCR.

Gene	5’-Primer (F)	3’-Primer (R)
ZO-1	ACGCTGGTGAAATCAAGGAAGAA	AGGGACATTCAACAGCGTGGC
Occludin	CAGGATGTGGCAGAGGAATACAA	CCTTGTCGTAGTCGCTCACCAT
Claudin-1	CACACGAGCTTTGATGGTGG	ACCAATGCTGACAAACCTGCAA
MUC2	ATGGAGAGCGTTGTGTTTGC	GTGAAGACCAGTTCGGGGAG
TLR4	CACCAGTTTCACTTCCCCTTGT	GCTTTGCTAGGGATGACCTCCAA
MyD88	GCTTATAGAAAGGAGGTGTCGG	TGAAAGTCGCATTCGTCGCT
NF-κB	ACAACGTCCTTCATTTAGCAA	TCTGATAAAGGTCGTTCCTCA
TNFα	TCAGATCATTCAGCGTCACC	GACACCATCACAAAGTTTCTGC
IL-6	GGTCATCCCAGATTCAGCTAC	CCCTCACGGTTTTCTCCATAA
NLRP3	CCAGCCTGAAGATCGGAGACCT	AGGAGCCACCCTAGAGGAGAGT
Caspase-1	CTATCCCATACTCTTGCCACG	TCCTTCACATCCACTTCAGC
ASC	CAGCATTCTGGATCGGCTCT	ATTTTCTCCTGCCTGATGCTT
IL-1β	TCATCTTCTACCGCCTGGAC	TAGCTTGTAGGTGGCGATGT
IL-18	ACCTCTGCCTCTATTTTGCTG	TTCAAAAGCTGCCATGTTCAG
GAPDH	TGATGCTCCCATGTTCGTGA	CTTTTCCCACAGCCTTAGCAG

F, forward; R, reverse

### Western Blot

Jejunum tissues were homogenized at 4°C in RIPA lysis buffer which contained protease inhibitors (PMSF) (Beyotime, Shanghai, China), and concentrations were determined using a BCA assay (35). Samples were further diluted, and 5× SDS-PAGE loading buffer was added and boiled for 5 min. Equal amounts of protein (10 μg) were loaded onto 12% SDS-polyacrylamide denaturing gels before being transferred to polyvinylidene fluoride (PVDF) membranes. After blocking with tris-buffered saline Tween (TBST) containing 5% non-fat milk powder for 1 h at room temperature, the membrane was incubated overnight with diluted primary antibodies against ZO-1 (1:1,000; ABclonal, Wuhan, China), occludin (1:1,000; Selleck Chemicals, USA), claudin-1 (1:1,000; ABclonal, China), TLR4 (1:500; Proteintech, Wuhan, China), MyD88 (1:500; Wanleibio, Shenyang, China), NF-κB (1:500; Wanleibio, China), NLRP3 (1:1,000; Wanleibio, China), ASC (1:500; Santa Cruz Biotechnology, Dallas, TX, USA), caspase-1 (1:500; Wanleibio, China), IL-1β (1:500; Wanleibio, China), IL-18 (1:1,000; Wanleibio, China), and GAPDH (1:5,000; Proteintech, China). Electrochemiluminescence liquid (ECL) (Tanon, Shanghai, China) was used to detect the signal. ImageJ software was used to assess protein levels. The target protein levels were normalized to GAPDH, and the radioactivity was compared with the control group.

### Immunofluorescence Analysis

MUC2 secretion was investigated using immunofluorescence analysis. A previous report provided detailed descriptions of the procedure (40). In brief, the prepared slides were incubated at 4°C for 16 h with the primary antibodies MUC2 (1:200; Service, Wuhan, China). The sections were then incubated for 1 h at 37°C with FITC-conjugated goat anti-rabbit Ig G (1:300; Service, Wuhan, China). Finally, the nucleus was stained for 10 min with DAPI. A fluorescence microscope was used to observe and capture the fluorescence patterns (Nikon, Japan).

### Statistical Analysis

The data were presented as the standard error of mean (SEM). Microsoft Excel 2016 and GraphPad Prism 8.0 (GraphPad Inc., La Jolla, CA, USA) was applied for data analysis and graphing. To compare differences with the control group, one-way ANOVA and multiple comparisons were used. The *P*-values of 0.05, 0.01, 0.005, and 0.001 were regarded as statistically significant.

## Results

### Cr(VI) Accumulation in the Jejunum and Its Effect on Growth Indexes and Histopathology

**Figure 1:**
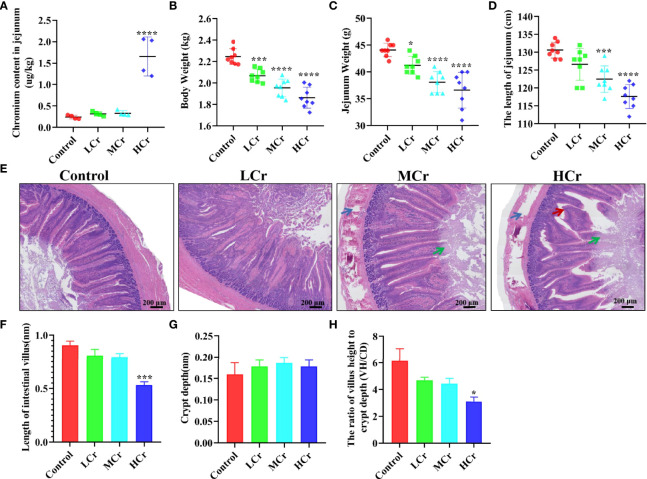
Cr(VI) exposure induced intestinal damage. **(A)** Cr(VI) content in jejunum tissues. **(B)** Body weight. **(C)** Jejunum weight. **(D)** The length of jejunum. **(E)** Histopathological variation in jejunum tissues (scale bar = 200 μm). **(F)** Length of intestinal villus. **(G)** Crypt depth. **(H)** The ratio of villus height to crypt depth (VH/CD). All data were presented as mean ± SEM; n ≥ 3 for each group. The symbol “*” denotes a statistically significant difference from the control group (*P < 0.05, ***P < 0.005 and ****P < 0.001).

As shown in **Figure 1A**, when compared to the control group, the Cr(VI) content in the jejunum tissues of the HCr group was significantly higher (*P <* 0.001). To investigate the effects of Cr(VI) exposure on intestinal injury, anatomical pathological and histological changes were identified. The changes in growth indexes are shown in **Figures 1B–D**, and the body weight in all Cr(VI)-treated groups was observably lower than in the control group (*P* < 0.005 or *P* < 0.001). The jejunum weight in all Cr(VI)-treated groups was observably lower compared with the control group (*P* < 0.05 or *P* < 0.001). The length of the jejunum in the MCr and HCr groups was noticeably shortened compared with the control group (*P* < 0.05 or *P* < 0.001). H&E staining revealed normal morphology, clear borders, and well-arranged epithelial cells in the control group. Nevertheless, the jejunum was injured in the MCr and HCr groups, with the shedding of the apical epithelium of the intestinal villi (green arrows) and destruction of the mucosal layer (blue arrows). In addition, we also observed intestinal villus breakage (red arrows) in the HCr group (**Figure 1E**). The length of the intestinal villus was significantly shorter in the HCr group compared with the control group (*P* < 0.005). The crypt depth (black arrows) was deepened (*P* > 0.05), while the ratio of villus height to crypt depth (VH/CD) in the HCr group was noticeably decreased compared with the control group (*P* < 0.05) (**Figures 1F–H**). The experiment data showed that Cr(VI) could lead to jejunum damage, thereby affecting the digestion and absorption of nutrients.

### The Effects of Cr(VI) Exposure on the Intestinal Barrier Function

**Figure 2:**
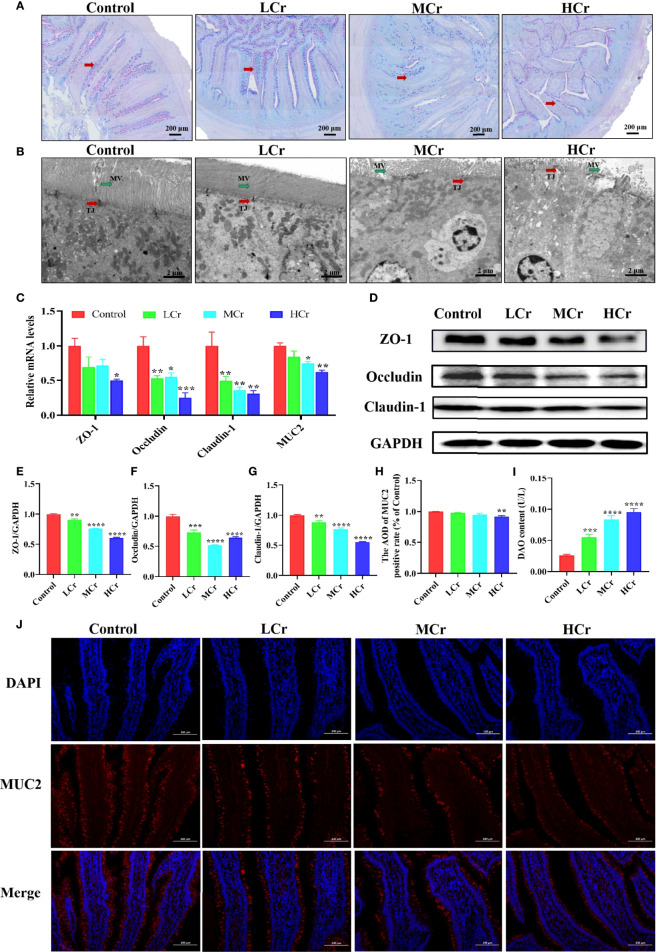
Effects of Cr(VI) exposure on intestinal epithelial barrier function. **(A)** PAS staining was used to determine the distribution of goblet cells (red arrows) in the intestine (scale bar = 200 μm). **(B)** Ultrastructure of the jejunum (scale bar = 2 μm). The red and green arrows represent changes in tight connections and microvilli, respectively. TJ stands for tight connection; MV stands for microvilli. **(C)** mRNA levels of genes related to the intestinal barrier. **(D–G)** Effects of Cr(VI) exposure on tight-junction protein expression levels in the duck jejunum. **(E)** ZO-1/GAPDH. **(F)** Occludin/GAPDH. **(G)** Claudin-1/GAPDH. **(H)** ImageJ analysis of MUC2 immunofluorescent staining results. **(I)** DAO content. **(J)** Immunofluorescence staining of MUC2 protein expression in jejunum tissue. The symbol “*” denotes a statistically significant difference from the control group (*P < 0.05, **P < 0.01, ***P < 0.005 and ****P < 0.001).

To explore whether Cr(VI) exposure could cause intestinal barrier damage, we examined the number of goblet cells (**Figure 2A**), the ultrastructural pathological changes (**Figure 2B**), and the mRNA and protein levels of intestinal barrier-related factors (**Figures 2C–G**). PAS staining showed that goblet cells reduced in a dose-dependent manner with increasing Cr(VI) concentration. TEM results showed that the microvilli of intestinal epithelial cells were shed, the number of microvilli was reduced, and the TJ structure of intestinal epithelial cells was damaged in the MCr and HCr groups. Compared with the control group, the ZO-1, occludin, claudin-1, and MUC2 mRNA levels were observably down-regulated in the HCr group (*P* < 0.05 or *P* < 0.01 or *P* < 0.005). Furthermore, the mRNA levels of occludin and claudin-1 in the LCr and MCr groups were significantly lower than those in the control group (*P* < 0.05 or *P* < 0.01) and the mRNA level of MUC2 in the MCr group was significantly lower than those in the control group (*P* < 0.05). Likewise, ZO-1, occludin, and claudin-1 protein levels were significantly down-regulated in all Cr(VI)-exposed groups compared to the control group (*P* < 0.01, *P* < 0.005, or *P* < 0.001). Meanwhile, MUC2 immunofluorescence results showed that Cr(VI) reduced the MUC2 fluorescence intensity (**Figure 2H, J**). DAO was also measured as a marker of intestinal epithelial cell maturity, integrity, and function, and the results revealed that DAO levels in serum were observably elevated in all Cr(VI)-exposure groups (*P* < 0.005 or *P* < 0.001) compared with the control group (**Figure 2I**).

### The Effects of Cr(VI) Exposure on Cytokines and Oxidative Stress Indices in the Jejunum

**Figure 3:**
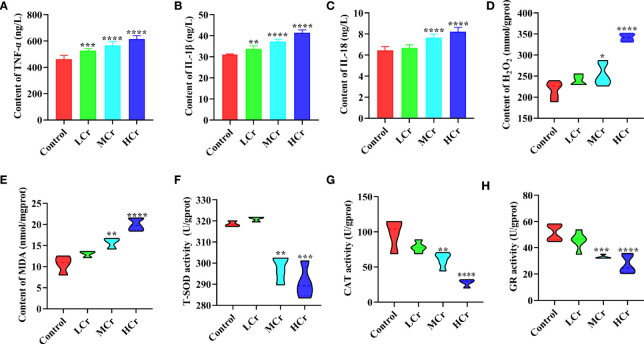
The effects of Cr(VI) on inflammation and oxidative stress in the jejunum. **(A)** TNF-α concentration. **(B)** IL-1β concentrations. **(C)** IL-18 concentrations. **(D)** H_2_O_2_ concentrations. **(E)** MDA concentrations. **(F)** T-SOD activity. **(G)** CAT activity. **(H)** GR activity. The symbol “*” denotes a statistically significant difference from the control group (*P < 0.05, **P < 0.01, ***P < 0.005 and ****P < 0.001).

The contents of cytokines in the jejunum are presented in **Figures 3A–C**. The TNF-α and IL-1β levels in jejunum were dramatically increased dose-dependently (*P* < 0.01 or *P* < 0.005 or *P* < 0.001) in all Cr(VI)-exposure groups in comparison with the control group. The level of IL-18 in the jejunum was observably elevated (*P* < 0.001) in the MCr and HCr groups compared with the control group. Additionally, oxidative stress indicators were measured in jejunum tissues to assess the extent of oxidative damage to ducks caused by Cr(VI) (**Figures 3D–H**). In comparison to the control group, H_2_O_2_ and MDA concentrations were observably elevated, while T-SOD, CAT, and GR activities were observably decreased (*P* < 0.01, *P* < 0.005, or *P* < 0.001) in the MCr and HCr groups.

### The Influence of Cr(VI) on the NF-κB Signaling Pathway in Jejunum Tissues

**Figure 4:**
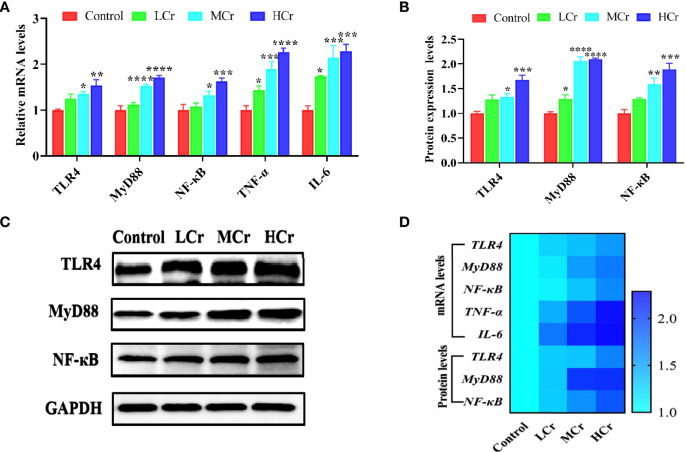
The influence of Cr(VI) on the NF-κB signaling pathway in jejunum tissues. **(A)** mRNA levels of genes involved in the NF-κB signaling pathway. **(B)** Quantitative analysis of NF-κB signaling pathway-related protein expression. **(C)** Western blot reveals that expression levels of NF-κB signaling pathway-related proteins. **(D)** A heatmap depicts the relationship between NF-κB signaling pathway-related mRNA and protein levels in jejunum tissues. The symbol “*” denotes a statistically significant difference from the control group (*P < 0.05, **P < 0.01, ***P < 0.005 and ****P < 0.001).

To determine whether Cr(VI) induced inflammation in jejunum tissues, we measured the mRNA and protein levels of NF-κB signaling pathway-related factors. TLR4, MyD88, NF-κB, TNF-α, and IL-6 mRNA levels were observably up-regulated in the MCr and HCr groups compared to the control group (*P* < 0.05, *P* < 0.01, *P* < 0.005, or *P* < 0.001), while TNF-α and IL-6 mRNA levels were observably up-regulated (*P* < 0.05) in the LCr group (**Figure 4A**). TLR4, MyD88, and NF-κB protein levels were also markedly up-regulated in the MCr and HCr groups compared to the control group (*P* < 0.05, *P* < 0.01, *P* < 0.005, or *P* < 0.001), and the protein level of MyD88 was markedly up-regulated (*P* < 0.05) in the LCr group (**Figures 4B, C**). The heatmap visually depicts the changes in these genes and proteins (**Figure 4D**). These findings suggested that Cr(VI) exposure activated the NF-κB signaling pathway in the jejunum, which was more pronounced in the HCr group.

### The Effects of Cr(VI) Exposure on the Activation of the NLRP3 Inflammasome in Jejunum Tissues

**Figure 5:**
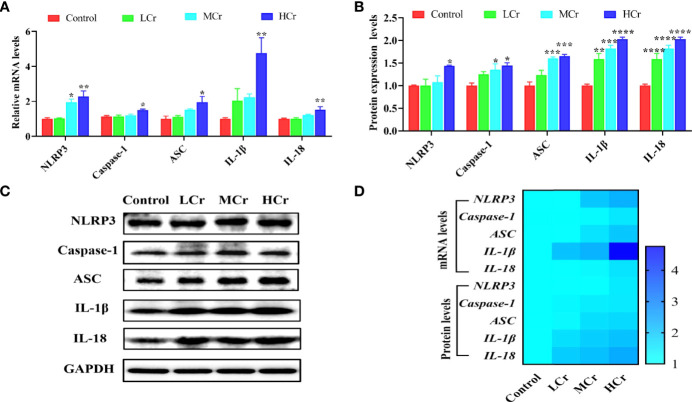
Effects of Cr(VI) exposure on NLRP3 inflammasome activation in jejunum tissues. **(A)** Pyroptosis-related gene mRNA levels. **(B)** Quantitative analysis of pyroptosis-related factor protein levels. **(C)** Western blot demonstrates pyroptosis-related protein expression. **(D)** A heatmap depicts the relationship between the expression of pyroptosis-related genes and proteins in jejunum tissues. The symbol “*” denotes a statistically significant difference from the control group (*P < 0.05, **P < 0.01, ***P < 0.005 and ****P < 0.001).

As shown in **Figure 5A**, NLRP3, caspase-1, ASC, IL-1β, and IL-18 mRNA levels were observably up-regulated in the HCr group compared with the control group (*P* < 0.05 or *P* < 0.01) and NLRP3 mRNA level was also observably up-regulated in the MCr group compared with the control group (*P* < 0.05). Simultaneously, NLRP3, caspase-1, ASC, IL-1β, and IL-18 protein levels were also observably up-regulated in the HCr group compared to the control group (*P* < 0.05, *P* < 0.005, or *P* < 0.001) and ASC, IL-1β, and IL-18 protein levels were observably up-regulated (*P* < 0.005 or *P* < 0.001) in the MCr group and IL-1β and IL-18 protein levels were observably up-regulated (*P* < 0.01 or *P* < 0.001) in the LCr group (*P* < 0.005) (**Figures 5B, C**). A heatmap was created to show the changes in these genes and proteins (**Figure 5D**). Experimental results indicated that Cr(VI) exposure activated the NLRP3 inflammasome in duck jejunum tissues.

## Discussion

Cr(VI) is a naturally occurring heavy metal that can be found in air, water, soil, and food. It is classified as highly toxic because it causes severe symptoms in humans and animals such as diarrhea, ulcers, eye and skin irritations, kidney dysfunction, and lung carcinoma (41, 42). As a result, many scientists have concentrated on the negative effects of Cr(VI) on human and animal health (43). Previous studies have found that long-term exposure to high concentrations of Cr(VI) in mice can lead to gastric mucosal bleeding, ulcers, weight loss, and severe effects on intestinal morphology and function (44, 45). However, its potential impact on the waterfowl intestine is unknown. We used a Cr(VI) poisoning model in this study to investigate the effects of Cr(VI)-induced jejunum damage in ducks and the underlying toxic mechanisms.

Intestines are crucial digestive organs, and damage to them can lead to nutrient absorption problems and an insufficient supply of nutrients to the body (46). In the current study, we noticed that the content of Cr(VI) was dramatically increased in the jejunum tissue. Additionally, the weight and length of the jejunum and the body weight of ducks were significantly decreased with increasing doses of Cr(VI) in the diet, which may be caused by damage to the intestinal epithelium. The epithelium of the small intestine is composed of abundant crypt–villus units. The intestinal villus is the crucial part in the increase in the mucosal surface area and then enhancement in the absorption of nutrients. Crypts are known as the home to a population of energetically reproducing epithelial cells, fueling the active intestinal epithelium. The VH/CD value was believed to be a sensitive indicator of the absorptive capacity of the small intestine (47). Therefore, changes in villus or intestinal gland anatomy have a direct impact on nutrient absorption. Accumulating evidence indicated that heavy metal exposure could cause shortening of intestinal villi, increase in recess depth and VH/CD values, and weight loss (48, 49). Similarly, Cr(VI) exposure resulted in Cr(VI) accumulation, shortening of the height of the jejunum gland, deepening in the crypt depth, and decrease in VH/CD values in the jejunum. These findings confirmed that continuous Cr(VI) accumulation in the jejunum caused significant damage to the intestinal epithelium, which may affect nutrient digestion and absorption in the ducks, resulting in poor growth performance.

Besides ingestion and absorption, the small intestine also has a barrier function that protects the body from hazardous substance such as toxins, pathogens, and foodborne antigens. MUC2 is the main protein component of the intestinal mucous layer that is secreted into the lumen of the large intestine from goblet cells in the epithelial lining. It forms a gel with small amounts of related mucins, forming an insoluble mucus barrier that protects intestinal epithelial cells (50). A previous study showed that by gavage of ducks with 80 mg/kg, goblet cells were significantly reduced, and at the same time, the mRNA and protein levels of MUC2 were also significantly reduced (22). Our results showed that with the increase in Cr(VI) concentration, the number of goblet cells was significantly reduced and the mRNA and protein levels of MUC2 were observably down-regulated. Tight-junction proteins are located between adjacent epithelial cells and play a crucial role in maintaining tight junctions in intestinal epithelial cells to form a physical barrier to enhance the intestinal protective function (51). DAO is a highly active intracellular enzyme in all mammalian intestinal mucosal epithelial cells, and DAO activity in serum can be used to assess the maturity, integrity, and functional status of intestinal epithelial cells (52). Once the barrier function was compromised, intestinal permeability increased, resulting in “leaky gut,” and DAO entered the bloodstream *via* the damaged mucosa, increasing the DAO levels in serum (53). Cr(VI) has been demonstrated to damage the intestinal epithelial TJ structure and decrease the levels of tight-junction proteins in mice by Zhu et al. (54). In the current study, we observed the effects of Cr(VI) on intestinal epithelial cell microvilli and TJ protein by using TEM and found that Cr(VI) exposure caused intestinal epithelial cell microvilli to fall off, the number of microvilli decreased, and the intestinal epithelial cell TJ structure was damaged. Meanwhile, the tight-junction proteins were detected, and the results indicated that Cr(VI) exposure decreased the mRNA and protein levels of ZO-1, occludin, and claudin-1 in the jejunum. To further verify the damage to the intestinal barrier, the level of DAO in serum was evaluated to check whether Cr(VI) exposure caused the “leaky gut”. Our result showed that Cr(VI) exposure increased the level of DAO in serum. These findings suggested that Cr(VI) exposure caused gut physicochemical barrier damage and increased jejunum permeability, resulting in more severe intestinal barrier destruction in ducks.

Substantial evidence suggests that intestinal epithelial barrier disruption and increased intestinal epithelial permeability increase exposure to bacteria and toxins, resulting in intestinal immune cell hyperstimulation and mucosal inflammation (55, 56). Furthermore, intestinal inflammation can cause intestinal epithelial barrier dysfunction and increased permeability (57). It has been reported that trichlorfon exposure decreased the expression levels of ZO-1, occludin, and claudin-2 in common carp accompanied by significantly increased expression levels of IL-1β and TNF-α (58). Moreover, high levels of TNF-α could disrupt the barrier function of Caco2 cells and increase intestinal permeability (59). IL-1β is a cytokine that has been linked to epithelial barrier dysfunction in the gut and has been shown to suppress the expression of claudin-3 in Caco2 cells (60). In a colitis model, IL-18 is critical in driving the pathological breakdown of intestinal barrier integrity; deletion of IL-18 conferred protection from colitis and mucosal damage in mice (61). In this study, TNF-α, IL-1β, and IL-18 concentrations were elevated with the increasing level of Cr(VI), which represented that Cr(VI) exposure induced inflammation in duck jejunum tissues. Many studies indicated that the production of pro-inflammatory factors is strongly related to oxidative stress (62). Thus, in the current study, we further investigated the redox state of the jejunum by measuring antioxidant enzymes and peroxidation products. T-SOD is a crucial antioxidant enzyme that converts superoxide O_2_^•-^ into H_2_O_2_, protecting the structure and function of cells from oxidative damage, H_2_O_2_ is then converted into water and oxygen *via* CAT catalysis (63, 64). Similarly, GR is important for detoxifying active metabolites and maintaining intracellular redox balance (65). Additionally, the accumulation of peroxidation product H_2_O_2_ could cause cell structure damage as well as gene damage and mutation. MDA is a by-product of lipid peroxidation that could promote cross-linking and polymerization of living macromolecules such as proteins and nucleic acids, resulting in cytotoxicity (66). Our results found that excessive dietary Cr(VI) significantly increased the contents of H_2_O_2_ and MDA and decreased the activities of SOD, CAT, and GR, which indicated that Cr(VI) exposure could induce oxidative stress in the jejunum of duck. In line with our results, Zhu et al. reported that excessive Cr(VI) could damage the intestine by inducing oxidative stress and inflammation (54).

The NLRP3 inflammasome has been shown to be a novel mechanism for intestinal inflammation, and it can be activated by ROS (67). The activation of the NLRP3 inflammasome triggers a series of immune responses, including the production of proinflammatory cytokines and chemokines and the recruitment of neutrophils and other immune cells, as well as cell death (68). However, activation of the NLRP3 inflammasome requires two steps: initiation and activation. TLRs recruit the signaling regulator MyD88 and a TIR domain-containing adaptor protein-induced interferon (TRIF) in the priming step to connect to signaling factors *via* NF-κB. This process promotes the transcription of NLRP3, pro-IL-1β, and pro-IL-18, as well as the production of multiple pro-inflammatory cytokines, including IL-1β, IL-6, and TNF-α (69). The inflammasome assembly step is triggered by several molecular and cellular events such as K+ efflux, Ca2+ signaling, and ROS generation. Procaspase-1 converts to cleaved-caspase-1 during the assembly of the NLRP3 inflammasome, promoting the cleavage of pro-IL-1β and pro-IL-18 precursors to IL-1β and IL-18 to induce inflammation (70, 71). Zhong et al. demonstrated that ATO exposure impaired intestinal barrier function and caused inflammatory injury in the duck jejunum by activating the LPS/TLR4/NF-κB signaling pathway (22). Similarly, Cr(VI) exposure increased the mRNA levels of TLR4, MyD88, NF-κB, TNF-α, and IL-6, as well as the protein expression levels of TLR4, MyD88, and NF-κB in jejunum tissues. Furthermore, Fan et al. demonstrated that zearalenone could activate the NLRP3 inflammasome *via* ROS, resulting in severe intestinal inflammation in mice. Our results also showed that Cr(VI) exposure elevated the mRNA and protein levels of NLRP3, ASC, caspase-1, IL-1β, and IL-18 in jejunum tissues. These findings suggested that Cr(VI), as a strong redox heavy metal, was involved in redox behavior and ROS generation, and induced jejunum inflammation by activating the NF-κB signaling pathway and the NLRP3 inflammasome. However, whether Cr(VI) induces intestinal barrier damage through ROS-mediated activation of the NF-κB signaling pathway and the NLRP3 inflammasome needs to be further verified by *in vitro* experiments.

## Conclusions

Overall, our studies revealed the negative impact on bioaccumulation, digestion, intestinal barrier function, antioxidant capacity, and immune response of the ducks following exposure to Cr(VI), further indicating that Cr(VI) induced intestine injury, oxidative stress, and immunotoxicity in ducks. Furthermore, the immunotoxicity of Cr(VI) was associated with the activation of the NF-κB signaling pathway and NLRP3 inflammasome by oxidative stress.

## Data Availability Statement

The original contributions presented in the study are included in the article/supplementary material. Further inquiries can be directed to the corresponding authors.

## Ethics Statement

The animal study was reviewed and approved by the animal ethics committee of Jiangxi Agricultural University (Approval ID: JXAULL-2022003).

## Author Contributions

GH, HC, and FY contributed to conception and design of the study. CX performed the statistical analysis and wrote the first draft of the manuscript. YL, JS, XY, YZ, and CW provided substantial contribution to experimental operation and data acquisition, wrote sections of the manuscript. FA, CZ, and YZ contributed to the grammatical revision of the manuscript. All authors contributed to manuscript revision, read, and approved the submitted version.

## Funding

This project was supported by the National Natural Science Foundation of China (No. 32060819; Beijing, P. R. China) awarded to GH.

## Conflict of Interest

The authors declare that the research was conducted in the absence of any commercial or financial relationships that could be construed as a potential conflict of interest.

## Publisher’s Note

All claims expressed in this article are solely those of the authors and do not necessarily represent those of their affiliated organizations, or those of the publisher, the editors and the reviewers. Any product that may be evaluated in this article, or claim that may be made by its manufacturer, is not guaranteed or endorsed by the publisher.
